# A Conserved Stem Loop Motif in the 5′Untranslated Region Regulates Transforming Growth Factor-β_1_ Translation

**DOI:** 10.1371/journal.pone.0012283

**Published:** 2010-08-26

**Authors:** Robert H. Jenkins, Rasha Bennagi, John Martin, Aled O. Phillips, James E. Redman, Donald J. Fraser

**Affiliations:** 1 Institute of Nephrology, School of Medicine, Cardiff University, Heath Park, Cardiff, Wales, United Kingdom; 2 School of Chemistry, Cardiff University, Park Place, Cardiff, Wales, United Kingdom; National University of Singapore, Singapore

## Abstract

Transforming growth factor-β_1_ (TGF-β_1_) regulates cellular proliferation, differentiation, migration, and survival. The human TGF-β_1_ transcript is inherently poorly translated, and translational activation has been documented in relation to several stimuli. In this paper, we have sought to identify *in cis* regulatory elements within the TGF-β_1_ 5′Untranslated Region (5′UTR). *In silico* analysis predicted formation of stable secondary structure in a G/C-rich element between nucleotides +77 to +106, and demonstrated that this element is highly conserved across species. Circular dichroism spectroscopy confirmed the presence of secondary structure in this region. The proximal 5′UTR was inhibitory to translation in reporter gene experiments, and mutation of the secondary structure motif increased translational efficiency. Translational regulation of TGF-β_1_ mRNA is linked to altered binding of YB-1 protein to its 5′UTR. Immunoprecipitation-RT-qPCR demonstrated a high basal association of YB-1 with TGF-β_1_ mRNA. However, mutation of the secondary structure motif did not prevent interaction of YB-1 with the 5′UTR, suggesting that YB-1 binds to this region due to its G/C-rich composition, rather than a specific, sequence-dependent, binding site. These data identify a highly conserved element within the TGF-β_1_ 5′UTR that forms stable secondary structure, and is responsible for the inherent low translation efficiency of this cytokine.

## Introduction

Transforming growth factor-β_1_ (TGF-β_1_) is a multifunctional cytokine involved in cellular proliferation, differentiation, migration, and survival. TGF-β_1_ regulates embryogenesis, angiogenesis, inflammation, and wound healing. The aberrant control of TGF-β_1_ is implicated in numerous pathological processes including tumorigenesis, atherosclerosis and fibrosis (reviewed in [1], [2]). Thus, understanding the regulation of TGF-β_1_ expression is of importance in homeostatic regulation and disease.

Tissue specific disparities in TGF-β_1_ mRNA and protein expression point to post-transcriptional regulation of synthesis [3]–[6]. Polysome analysis confirms that TGF-β_1_ is inherently poorly translated, in cultured cells and in mouse liver [7], [8]. The TGF-β_1_ mRNA transcript possesses a long 5′ untranslated region (UTR) of approximately 867-nucleotides and a 3′UTR of 137-nucleotides, both of which are highly G/C-rich, features suggestive of translational control [9]. Reporter gene experiments demonstrate that the proximal 5′UTR inhibits translation while in similar experiments the 3′UTR appears stimulatory [8]. Furthermore, deletion analysis and *in vitro* translation suggest that translation inhibition by the 5′UTR is primarily due to a limited pool of trans-acting factors interacting with the 5′UTR [8]. We have previously investigated translational control of TGF-β_1_ in renal Proximal Tubular Epithelial Cells. Our data show specific translational activation of TGF-β_1_ in response to a number of stimuli, including platelet-derived growth factor [10], [11] and TGF-β_1_ itself [12]. Recently, we have investigated protein binding to the TGF-β_1_ 5′UTR. Two protein complexes were detected, of approximate molecular weight 50 kDa and 100 kDa, in which the multifunctional DNA/RNA binding protein Y-Box Binding Protein-1 (YB-1) was a major constituent [13]. YB-1 was detected in association with TGF-β_1_ mRNA and, following translational activation, association of YB-1 with TGF-β_1_ mRNA was reduced [13].

It is therefore apparent that post-transcriptional regulation of TGF-β_1_ synthesis is of general importance, and that a major translation control element may reside in the 5′UTR. The G/C-rich nature of the 5′UTR has led to the proposal that secondary structure within this area may be relevant for its inherently poor translational efficiency [14], but this has not been formally tested. The aim of this study was to identify sequence within the TGF-β_1_ 5′UTR responsible for translational inhibition, and to examine the interaction of YB-1 with elements identified. Our data identify a highly conserved element within the TGF-β_1_ 5′UTR that forms stable secondary structure, and is responsible for the inherent low translation efficiency of this cytokine.

## Results

### Analysis of TGF-β_1_-5′UTR mRNA secondary structure

**Figure 1:**
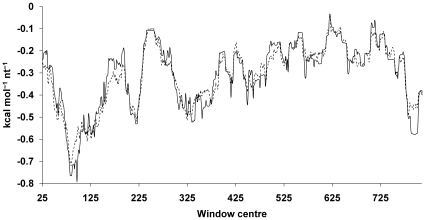
Ensemble free energy per nucleotide *vs.* window centre. Ensemble free energy per nucleotide *vs.* window centre is plotted for 50 nucleotide sliding windows (solid line) of the TGF-β_1_ 5′UTR. Mean ensemble free energy of 100 scrambled windows *vs.* window centre is plotted (dashed line).

The TGF-β_1_ 5′UTR was investigated computationally. Local secondary structure was predicted using the RNAfold software [15], [16] to study sliding windows of the sequence ranging from 30 to 100 nucleotides in length. In addition to determining the minimum free energy structure and ensemble free energy via a partition function calculation [17], the significance of any secondary structure was assessed by calculation of the segment score [18] in which a comparison was made against a collection of scrambled sequences, which preserve the dinucleotide frequencies of each window. As suggested by Workman and Krogh [19] the choice was made to conserve dinucleotide frequencies due to bias in the dinucleotide distribution of eukaryotic genomes [20]. A plot of the ensemble free energy per nucleotide against the location of the window centre (Figure 1) shows a cluster of minima located between nucleotides +80 to +100, suggesting that this will be the region of the UTR with the most stable secondary structure. A maximum centred around nucleotide 620 indicates that this region may show a propensity for an absence of structure. It is notable that a plot of the mean ensemble free energy of the scrambled sequences exhibits minima and maxima in the same locations, indicating that these extrema arise primarily due to the nucleotide composition of the sequence in these regions. Closer examination of the 80 nucleotides starting at +59, which spans the cluster of minima, reveals a balanced G/C-rich composition of 36G 36C 4A 4U, in keeping with an intrinsic bias towards formation of stable secondary structure in this region. The segment scores also exhibited a negative spike in this region, but the occurrence of such spikes is to be expected given the length and nucleotide composition of the 5′UTR, and indeed spikes with similar amplitude and frequency were observed when randomly scrambled sequences of the same dinucleotide composition as the 5′UTR were subjected to identical segment score calculations (data not shown).

**Figure 2:**
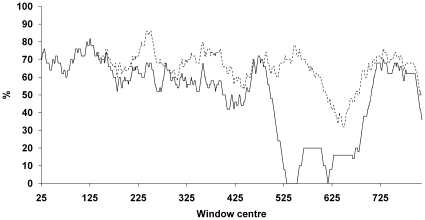
Percentage of bases paired with probability >0.5 in 50 nucleotide windows of the TGF-β_1_ 5′UTR. Individual base pairs with probability >0.5 (solid line) and sum of probabilities over all pairings >0.5 (dashed line) are plotted.

The sequence of the TGF-β_1_ 5′UTR was examined with RNAfold, and the minimum free energy and centroid structure visualised. The centroid structure depicts individual base pairs with probability >0.5, to highlight those pairs most likely to form, regardless of whether or not they exist in the single minimum free energy structure. When the pairing probabilities are expressed as the percentage of paired bases in each 50 nucleotide window (Figure 2) it is apparent that the RNA is predicted to assume particularly well defined secondary structure towards its proximal end, and that there is a poorly structured region between nucleotides +500 to +700. The segment scores calculated for the 5′UTR as a whole using minimum and ensemble free energies are +1.1 and +0.8 respectively, indicating that there is no more or less structure to the UTR than is dictated by its G/C-rich nature. Our observations are consistent with the conclusions of the wider study of mRNA secondary structure by Workman and Krogh [19] which also found no evidence that computed folding free energies of mRNA were lower than those of scrambled sequences with conserved dinucleotide frequencies.

**Figure 3:**
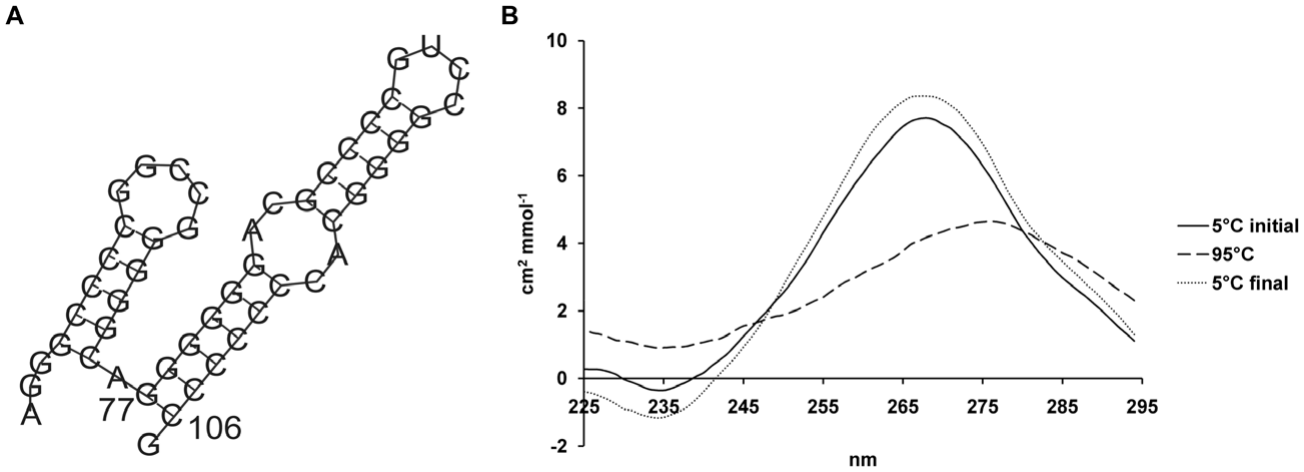
*In silico*- and *In vitro-* analysis of nucleotides 77–106. **A**. Centroid structure of the G/C-rich stem loop predicted between nucleotides 77–106 of the TGF-β_1_ 5′UTR. **B**. Circular dichroism spectra (Δε) of the TGF-β_1_ 5′UTR nucleotides 75–113 (7µM) in 10 mM Tris.HCl, pH 8, 100 mM KCl, 6 M urea. (i) solid line, initial spectrum at 5°C; (ii) dashed line, 95°C; (iii) dotted line, final spectrum at 5°C.

The RNAfold centroid structure of the TGF-β_1_ 5′UTR exhibits a G/C-rich region predicted to form a stem loop between nucleotides +77 to +106 (Figure 3A) for which base pair probabilities were calculated to be >0.9, coinciding with the region predicted to adopt the most stable secondary structure in the windowed calculations. In order to test for secondary structure formation in this region, circular dichroism spectroscopy was performed on an oligoribonucleotide comprising nucleotides +75 to +113 of the TGF-β_1_ 5′UTR. Under non-denaturing conditions at 20°C the oligoribonucleotide exhibited a positive peak at 269 nm, which did not display a melting transition up to a temperature of 95°C (data not shown). However, upon heating in 6 M urea, a sharp decrease in ellipticity at 269 nm was observed above 80°C, accompanied by a shift of the maximum to 276 nm (Figure 3B) and a hyperchromic effect in the absorbance at 260 nm. These spectral changes are consistent with thermal melting of a stem loop and unstacking of bases, and were reversed upon cooling the sample to 5°C (Figure 3B). Sequences with runs of consecutive guanines have the additional possibility of forming G-quadruplex structures, which are not considered by RNAfold. However, the characteristic hypochromic effect [21], [22] at 295 nm upon melting of G-quadruplex was absent which leads us to conclude that a quadruplex is not a major conformation of this oligoribonucleotide.

### Species Conservation of the Stem Loop Secondary Structure

**Figure 4:**
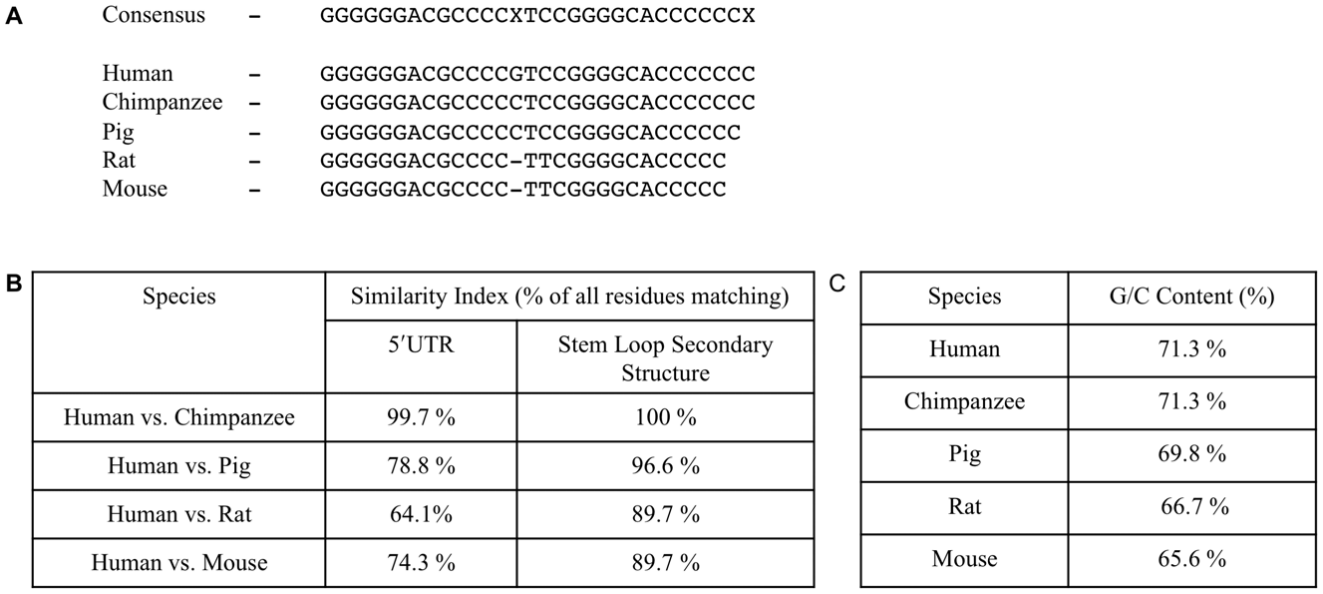
Sequence alignment of TGF-β_1_ 5′UTR. TGF-β_1_ mRNA reference sequence accession numbers for human (NM_000660), chimpanzee (XM_512687 - derived from automated computational analysis using gene prediction method GNOMON, www.ncbi.nlm.nih.gov/sites/entrez), pig (NM_214015), rat (NM_021578 - extended by comparison of overlapping ESTs, X52498, CB806681, EX490293, and CO573793), and mouse (NM_011577). Dashes represent gaps in the alignment. **A**. Multiple sequence alignment of the TGF-β_1_ 5′UTR stem loop secondary structure by the Clustal W method (DNASTAR® Lasergene 7.2). **B**. Pairwise sequence alignment of both the entire TGF-β_1_ 5′UTR and the stem loop secondary structure by the Wilbur-Lipman method (DNASTAR® Lasergene 7.2). **C**. Percent C/G composition of the 5′UTRs.

The human TGF-β_1_ 5′UTR was compared to the available full-length sequences from chimpanzee, pig, rat, and mouse (Figure 4). Initially, the sequences were subjected to Clustal W multiple sequence alignment generating a consensus sequence (Figure 4A). Further analysis was performed by Wilbur-Lipman pair-wise sequence alignment, comparing the human sequence to each species individually. In the species analysed the 5′UTR demonstrates a high degree of conservation, between 64.1–99.7% similarity (Figure 4B), using a threshold for conservation of 70% similarity with mouse over 100-nucleotides [23]–[27]. This increases to 89.7–100% similarity when only comparing the sequence of the stem loop secondary structure (Figure 4B) suggesting that the stem loop secondary structure identified between nucleotides +77 to +106 is evolutionarily conserved.

### Translational Inhibition by the TGF-β_1_ 5′UTR

**Figure 5:**
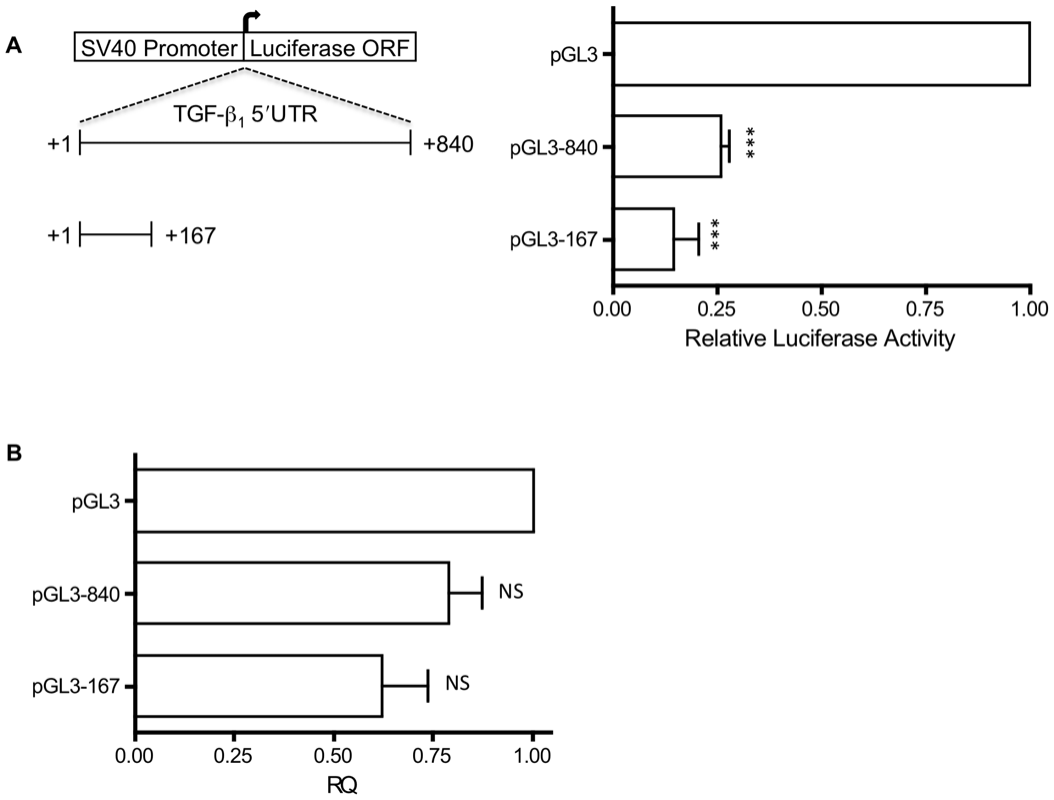
A. Reporter gene analysis of the TGF-β_1_ 5′UTR. Luminescence was measured by Dual Luciferase Reporter Assay Kit (Promega). Luciferase activity is expressed as normalised firefly luciferase activity relative to that of the pGL3-Control vector. Results shown represent mean (±SEM) of four experiments performed in triplicate. **B**. Quantification of luciferase mRNA by RT-qPCR according to standard protocol using SYBR® Green dye. Results shown represent mean (±SEM) of four experiments performed in triplicate. Statistical analysis was performed using an unpaired *t* test with Welch's correction (P<0.005***).

In order to confirm the translation inhibitory nature of the TGF-β_1_ 5′UTR two heterologous luciferase reporter constructs were generated, containing fragments of the 5′UTR corresponding to nucleotides +1 to +167 (pGL3-167) and nucleotides +1 to +840 (pGL3-840). The relative luciferase activity of both the pGL3-167 and pGL3-840 vectors was significantly reduced, approximately 5-fold and 3-fold respectively, compared to the pGL3-Control empty vector (Figure 5A). The changes in relative luciferase activity appeared to be predominantly post-transcriptional, as there was no significant difference in the quantities of mRNA transcripts produced by the individual vectors, as assayed by RT-qPCR (Figure 5B).

### Mutational Analysis of the Translational Inhibitory TGF-β_1_ 5′UTR

**Figure 6:**
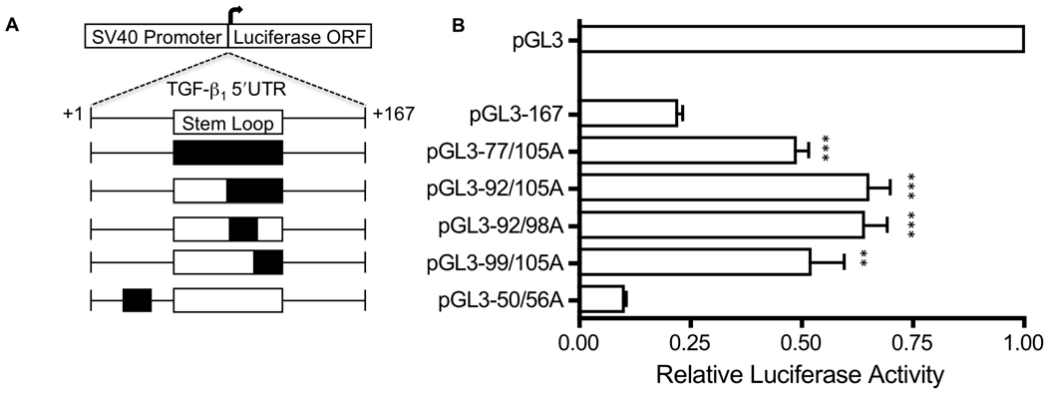
Reporter gene analysis of TGF-β_1_ 5′UTR mutations. **A**. Schematic diagram of the TGF-β_1_ 5′UTR endogenous and mutant 167-nucleotide fragments as described in the methods. Open boxes indicate the stem loop secondary structure, filled boxes indicated the position of the adenine residue substitutions. **B**. Luminescence was measured by Dual Luciferase Reporter Assay Kit (Promega). Luciferase activity is expressed as normalised firefly luciferase activity relative to that of the pGL3-Control vector. Results shown represent mean (±SEM) of four experiments performed in triplicate. Statistical analysis was performed using an unpaired *t* test with Welch's correction (P<0.05**, P<0.005***).

The above results confirm the overall net translational inhibitory nature of the TGF-β_1_ 5′UTR, and are in keeping with the presence of a major inhibitory element within nucleotides +1 to +167. A series of mutant luciferase reporter constructs were generated, containing the +1 to +167 nucleotide fragment of the TGF-β_1_ 5′UTR, incorporating mutations within the stem loop sequence (Figure 6A). Mutational, rather than deletional, analysis was performed to maintain sequence length, in view of the potential for influence of translational efficiency by 5′UTR leader length. Adenine substitution was chosen to avoid spurious enhanced YB-1 protein binding [28].

The effect of the mutations on the secondary structure was assessed computationally by folding each sequence with RNAfold. As intended by design, mutated regions were universally found to be unpaired. The pGL3-77/105A and pGL3-92/105A mutants are devoid of all base pairs with probability >0.5 between nucleotides +77 to +105, indicative of abolition of structure in this region of the RNA. The centroid structure of the pGL3-99/105A mutant reveals the possibility for formation of an alternative stem loop involving the remaining endogenous nucleotides. The pGL3-92/98A mutant likewise shows an alternative helix in the centroid structure in which nucleotides +70 to +74 are paired with +102 to +106. However, these secondary structures are considerably less favourable than the endogenous stem loop secondary structure, with calculated ensemble free energies of folding 9.8 and 12.7 kcal/mol higher than the endogenous sequence.

Complete abolition of the stem loop secondary structure in the mutants pGL3-77/105A and pGL3-92/105A resulted in a reversal of translational inhibition as assayed by an increase in relative luciferase activity compared to the endogenous sequence pGL3-167 (Figure 6B). Furthermore, even partial changes in secondary structure in the mutants pGL3-92/98A and pGL3-99/105A relieved the translational inhibition (Figure 6B). However, a control mutation at nucleotides +50 to +56 in pGL3-50/56A did not relieve translational inhibition (Figure 6B).

### Protein binding to TGF-β_1_ mRNA

**Figure 7:**
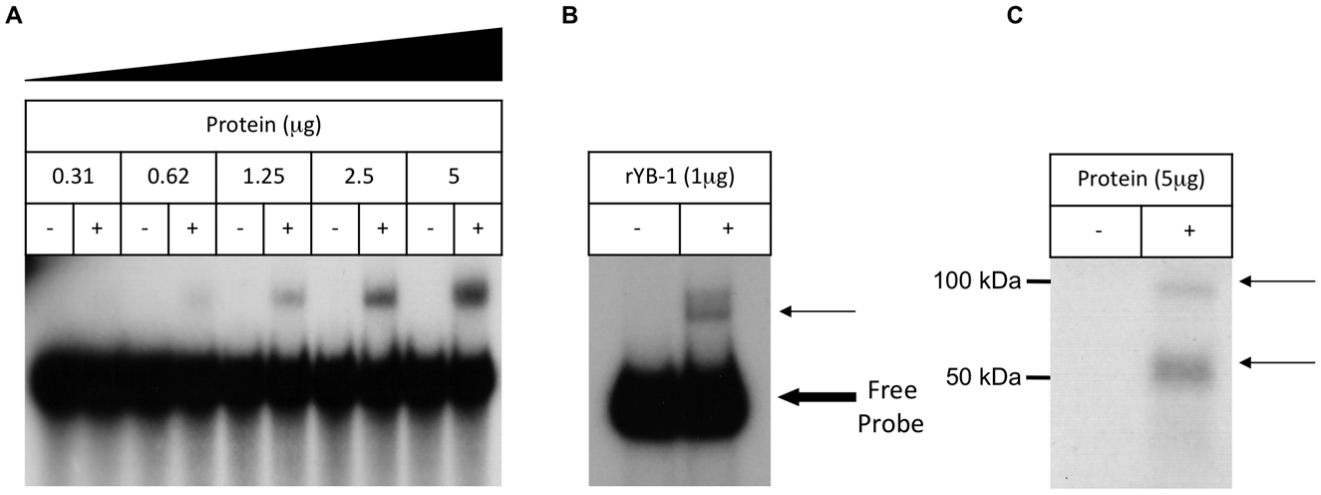
Protein binding to the TGF-β_1_ 5′UTR. Protein binding to the first 167 nucleotides of the TGF-β_1_ 5′UTR examined by RNA-EMSA and UV-Crosslinking. **A**. RNA-EMSA of a two-fold dilution of HK-2 cell protein binding to the 167-nucleotide transcript. **B**. RNA-EMSA of 1 µg of recombinant YB-1 protein binding to the 167-nucleotide transcript. C. UV-Crosslinking of 5 µg of HK-2 cell protein binding to the 167-nucleotide transcript. The bold arrow indicates the non-retarded free probe and the thin arrow indicates the retarded bands. The results are representative of four individual experiments.

**Figure 8:**
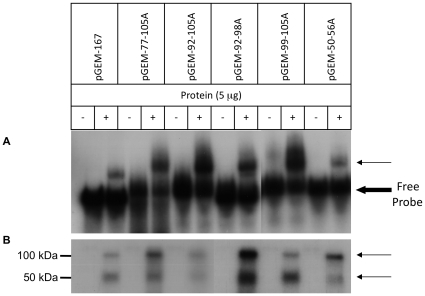
Protein binding to TGF-β_1_ 5′UTR mutations. The mutant 167-nucleotide transcripts are as described in the methods and depicted in Fig. 6A. **A**. RNA-EMSA. **B**. UV-Crosslinking. The bold arrow indicates the non-retarded free probe and the thin arrow indicates the retarded bands. The results are representative of four individual experiments.

Taken together, the above data identify an evolutionarily conserved G/C-rich element in the TGF-β_1_ 5′UTR that forms a stable stem loop secondary structure, and is inhibitory to translation. The work of Allison *et al.* demonstrated that the translational inhibitory action of the TGF-β_1_ 5′UTR is dependent on a limited pool of trans-acting factors [8]. Previous work from our laboratory showed that protein complexes bind the TGF-β_1_ 5′UTR and that these incorporate YB-1 [13]. Protein binding to endogenous and mutated sequences was therefore investigated. Electrophoretic Mobility Shift Asssay (EMSA) performed with the endogenous 167-nucleotide transcript and whole cell protein extracts resulted in the formation of a retarded band (Figure 7A). Recombinant YB-1 was capable of reproducing this binding interaction (Figure 7B). This is in contrast to the two retarded bands seen in our previous work [13]. This is attributed to the inclusion of poly I∶C in the EMSA binding reaction to increase the stringency of the assay. When EMSAs were performed in the absence of poly I∶C, two bands were detected (not shown). UV-Crosslinking experiments detected two protected bands under denaturing conditions with molecular weights of approximately 50 and 100 kDa (Figure 7C), confirming previous observations [13]. Previous comparative analysis of the TGF-β_1_ 5′UTR identified two putative YB-1 binding sites. RNA oligonucleotides homologous to these putative binding sites were able to compete with the proximal TGF-β_1_ 5′UTR for YB-1 binding, suggesting that YB-1 formed complexes at these loci [13]. However, EMSAs utilising mutations of both putative binding sites failed to demonstrate any change in YB-1/cellular protein binding (Figure 8). Similarly, UV-Crosslinking identified two protected RNA elements, consistent with our previous work, but no differences were seen utilising probes incorporating mutant 5′UTR sequences (Figure 8).

**Figure 9:**
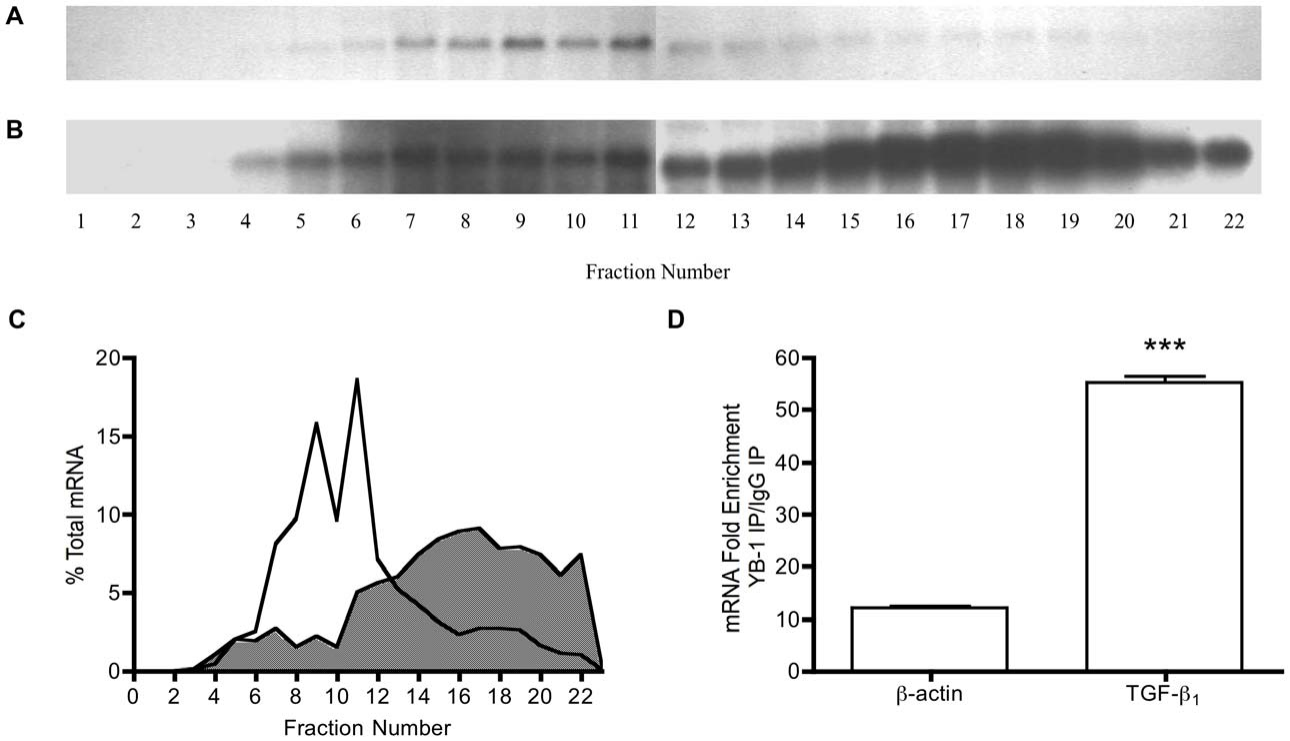
Comparison of translationally active and repressed mRNA. Northern blot of fractionated mRNA probed for **A**. TGF-β_1_ and **B**. β-actin as described in the materials and methods. **C**. Graphical representation of polysome distribution of β-actin **(shaded area)** and TGF-β_1_ **(solid line)**, expressed as a percentage of total mRNA detected on a given blot in each fraction. **D**. Immunoprecipitation of YB-1/mRNA complexes and RT-qPCR for β-actin and TGF-β_1_. The results are representative of four individual experiments and where appropriate represent mean (±SEM) performed in triplicate. Statistical analysis was performed using an unpaired *t* test with Welch's correction (P<0.005***).

YB-1 has general RNA binding activity, facilitated by high G/C content. The above data is suggestive that YB-1 binds to the TGF-β_1_ 5′UTR dependent on its G/C-rich nature, rather than by recognition of specific binding sites. We have recently detected association of YB-1 protein with TGF-β_1_ mRNA by Immunoprecipitation followed by RT-PCR [13]. In these previous experiments, activation of TGF-β_1_ translation was associated with decreased binding of YB-1 to endogenous TGF-β_1_ mRNA, suggesting a functional link between low basal efficiency of TGF-β_1_ translation, and high YB-1 binding [13]. In order to ascertain whether more YB-1 binds to TGF-β_1_ mRNA than to other, well-translated mRNA, comparison of YB-1 binding to endogenous transcripts was made by immunoprecipitation-RT-qPCR (IP-RT-qPCR). β-actin was chosen as a comparator, as it is a ubiquitously highly expressed protein product of a primary transcript without evidence of alternative splicing, and without features suggestive of translational control in its UTRs. Translational activity of endogenous TGF-β_1_ and β-actin transcripts was first compared by polysome analysis. TGF-β_1_ mRNA was localised primarily in fractions 4–12 of polysome preparations, reflecting its predominant association with free-mRNPs and monosomes (Figure 9A and C). In comparison the β-actin transcript was predominantly localised in fractions 12–22, demonstrating its polysome association, and high translational activity (Figure 9B and C). Next, binding of YB-1 to endogenous TGF-β_1_ and β-actin transcripts was examined by IP-RT-qPCR. IP of mRNA/YB-1 protein complexes revealed a five-fold enrichment of YB-1 on the TGF-β_1_ transcript in comparison to the β-actin (Figure 9D). Taken together, this data confirms that YB-1 binds preferentially to the TGF-β_1_ transcript, and is suggestive that this may relate to high G/C content and UTR length, rather than interaction with a specific binding locus.

## Discussion

We have identified an evolutionarily conserved motif in the TGF-β_1_ 5′UTR located between nucleotides +77 to +106 that forms a stable stem loop secondary structure and is responsible for the translational inhibitory nature of the transcript. Translation can be modulated by various characteristics of the 5′UTR including length, translation start-site context, secondary structure, binding sites for regulatory proteins, upstream open reading frames (uORFs) and internal ribosome entry sites (IRES) [29]. Translational regulation by secondary structure is partially dependent on stability and position. Moderately stable structures (−30 kcal/mol) within approx. 40 nucleotides of the 7-methylguanosine (m^7^G) cap structure block access of the 43S pre-initiation complex [30] and initiation factors eIF4A and eIF4B [31], [32] to reporter mRNA. When the same secondary structure is positioned further downstream, the 43S pre-initiation complex binds freely to the m^7^G cap structure, enabling 40S ribosome scanning and translational initiation [30], [33]. However, a more stable secondary structure (−50 kcal/mol) in the same position presents an impenetrable barrier to 40S ribosome scanning [30], [33]. The position of the stem loop secondary structure in the TGF-β_1_ 5′UTR suggests that it is most unlikely to act via steric hindrance of 43S pre-initiation complex binding to the m^7^G cap structure. Interestingly, the stem loop secondary structure is predicted to be moderately stable (−24 kcal/mol) suggesting that it would not in itself abrogate 40S scanning, and that it may require the cooperative action of other regulatory factors to cause the inherently low translational efficiency of TGF-β_1_.

Our previous work demonstrated the physical and functional interaction of the major RNA binding protein YB-1 with the TGF-β_1_ 5′UTR [13]. YB-1 is a major constituent of translationally inactive messenger ribonucleoprotein particles (mRNPs) [40]–[42] and is also present in active polysomes [34], [35] where it is required for translation initiation [37]. YB-1 appears to display a concentration-dependent effect on global translation. Low YB-1/mRNA ratios typical for polysomal mRNA may result in translational stimulation, whereas high ratios are typical for a translationally repressed mRNA [34]–[36] and result in translational inhibition via displacement of the translation initiation factor eIF4G [38], [39]. Additionally, YB-1 has been shown to displace eIF4E and eIF4A, which results in stabilisation of the mRNA [40] and modulates the PABP-stimulatory activity of eIF4F and assembly of ribosome initiation complexes [41]. Several specific regulatory interactions of YB-1 with mRNA transcripts have also been reported, including Interleukin-2 [42], Granulocyte-Macrophage Colony-Stimulating Factor [43], and YB-1 itself [44], [45]. In our previous work, we found that YB-1 silencing with siRNA prevented *de novo* TGF-β_1_ synthesis [13]. Additionally, activation of TGF-β_1_ translation was associated with decreased YB-1/TGF-β_1_ mRNA association and, while enforced expression of YB-1 did not alter basal TGF-β_1_ production, it did prevent translational activation of TGF-β_1_ by stimuli such as Platelet Derived Growth Factor [13]. Thus, it is clear that YB-1 has specific effects on translational efficiency of transcripts including TGF-β_1_.

Our previous polysome analysis shows that TGF-β_1_ mRNA is monosome-associated, and moves to polysomes in response to specific translationally activating stimuli, consistent with translational regulation at the level of initiation [10]–[12]. Translational activation of TGF-β_1_ is associated with decreased binding of YB-1 to the TGF-β_1_ transcript [13]. However, mutation of the stem loop in the TGF-β_1_ 5′UTR de-repressed translation without apparent alteration in YB-1 binding. YB-1 has complex and sequence-specific effects on the thermodynamic properties of RNA secondary structure [46], displays a higher affinity for single stranded nucleic acid sequences [46]–[48], and preferentially binds G/C-rich transcripts [49]. Furthermore, YB-1 binding may alter the kinetics of strand exchange, accelerating the rate of optimal duplex formation [50]. It is therefore possible that YB-1 may bind to the 5′UTR with relatively high affinity based on the G/C content of the UTR and the presence of secondary structure, rather than on specific sequence, and may cooperate with the stem loop to inhibit TGF-β_1_ translation by facilitating duplex formation. Given the ubiquitous nature of YB-1, and its widespread roles in the regulation of global translation and stability of transcripts, it is likely that additional regulatory factors are involved in its specific actions with respect to TGF-β_1_. One such factor is DDX3, an RNA helicase that remodels the 5′UTR during translation, recently shown to associate with the TGF-β_1_ 5′UTR [51].

In summary, in this paper we have identified a highly conserved *in cis* regulatory element within the TGF-β_1_ 5′UTR responsible for its inherently low translational efficiency. The mechanisms by which this element, together with YB-1 and other regulatory factors, control TGF-β_1_ synthesis are an important area for further study.

## Materials and Methods

### Materials

All general reagents were purchased from Sigma-Aldrich (Poole, UK), Promega (Southampton, UK), New England Biolabs (MA, USA), and Invitrogen (Paisley, UK) unless stated otherwise. Oligonucleotides were purchased from ThermoFisher Scientific (MA, USA). Radioisotopes were purchased from Perkin Elmer (Buckinghamshire, UK).

### Antibodies and Recombinant Protein

Recombinant YB-1 and a monoclonal anti-YB-1 antibody (raised to the *N*-terminus) were a kind gift from Dr PR Mertens, Division of Nephrology and Clinical Immunology, University Hospital RWTH-Aachen, Aachen, Germany. A polyclonal anti-YB-1 antibody was purchased from Abcam (Cambridge, UK).

### Computational Analysis of the TGF-β_1_ 5′ UTR

RNA secondary structure prediction was performed using the Vienna RNA package version 1.7.2 [15], [16]. Folding of individual sequences was performed using the stand-alone RNAfold program with the default minimum free energy algorithm, or with the –p option to calculate the partition function and base pairing probability matrix [17]. 200 randomly scrambled TGF-β_1_ 5′UTR sequences with conserved dinucleotide frequencies were generated using the squid utilities version 1.9g StrDPShuffle function [52]. Segment scores (*S*) were calculated for both minimum and ensemble free energies according to equation 1 [18], where *E* is the free energy for folding the endogenous sequence, and *E*_scrambled_ and *σ* are the mean and standard deviation of the folding free energy of the scrambled sequences.
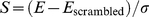
(1)Folding of windowed sequences was performed using the Vienna RNAlib library functions called from our own C code. Scores were calculated for windows of the TGF-β_1_ 5′UTR sequence according to a method based on that of Le and Maizel [18]. A window was slid along the sequence in the 5′ to 3′ direction in single nucleotide steps, and the window size was increased from 30 to 100 nucleotides in increments of 2. Each window was folded to obtain minimum and ensemble free energies. For each window, 100 randomly scrambled sequences with conserved dinucleotide frequencies were generated with the StrDPShuffle function, and segment scores calculated according to Equation 1. It was verified that the scrambled sequences were free from duplicates for windows of length 30 nucleotides or greater. To assess the significance of the results the same procedure was performed for 50 nucleotide sliding windows of 100 sequences constructed by scrambling the entire TGF-β_1_ 5′-UTR with conservation of dinucleotide frequencies prior to application of the sliding window.

### Cell Culture and Protein Extraction

HK-2 cells are human renal proximal tubular epithelial cells (PTC) immortalised by transduction with human papilloma virus E6/E7 genes [52]. HK-2 cells were purchased from American Type Culture Collection (Middlesex, UK) and cultured in DMEM/Ham's F12 supplemented with 10% FCS (Biological Industries Ltd, Cumbernauld, UK), 2 µM L-glutamine, 20 mM HEPES, 5 µg/ml insulin, 5 µg/ml transferrin, 40 ng/ml hydrocortisone and 5 ng/ml sodium selenite. Cells were grown at 37°C in 5% CO_2_ and 95% air. The growth medium was replenished every 3–4 days until confluent. With the exception of the cells used for transfection, cells were growth arrested in serum-free medium for 48 h before use in experiments. All experiments were performed in serum-free conditions. Total cell protein extracts were performed as described previously [13]. In all aspects of cell biology that we have studied previously, HK-2 cells respond in an identical manner to primary cultures of human PTC [6], [10], [11], [13], [53]–[56]. They are therefore a good model from which general conclusions can be drawn, in terms of proximal tubular cell biology.

### Plasmid Construction

**Figure 10:**
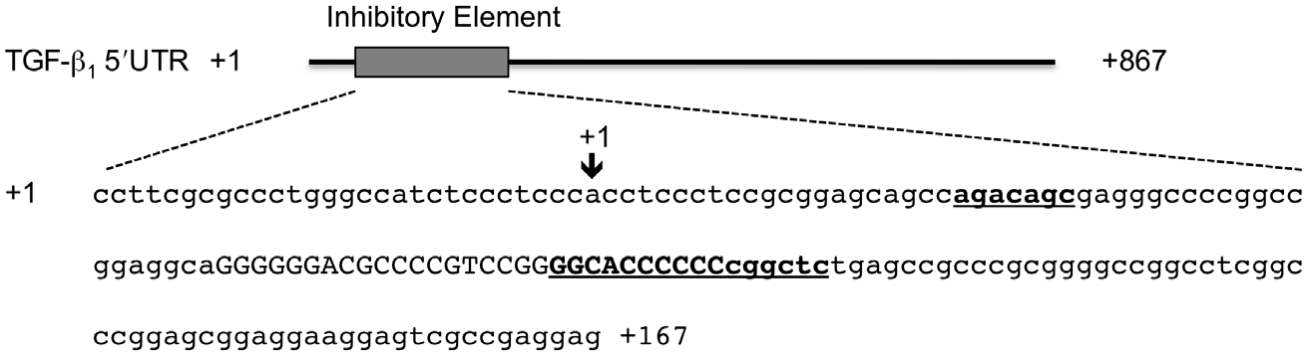
Diagrammatic representation of the TGF-β_1_ 5′UTR inhibitory element. The TGF-β_1_ 5′UTR inhibitory element nucleotides +1 to 167 derived from the reference sequence: accession number NM_000660. The sequence in capitals identifies the G/C-rich stem loop secondary structure and the bold/underlined sequence indicates the previously identified putative YB-1 binding sites. The arrow indicates the start position of the numbering convention of Kim et al [14].

Luciferase reporter constructs were generated containing inserts of the TGF-β_1_ 5′UTR corresponding to nucleotides +1 to +167 (pGL3-167) and nucleotides +1 to +840 (pGL3-840) (Figure 10). The numbering convention refers to the published reference sequence for the TGF-β_1_ mRNA transcript: accession number NM_000660. cDNA was generated as previously described [10]. The 167 and 840 nucleotide inserts were amplified using specific primers (Table 1), which incorporated the restriction sites *Hind*III and *Nco*I. The resultant inserts were digested with *Hind*III and *Nco*I and cloned into *Hind*III/*Nco*I digested pGL3-Control vector (Promega). The NcoI site contains the initiation ATG of firefly luciferase thereby excluding any vector-derived 5′UTR sequence.

**Table 1:** Oligonucleotide primer sequences, given in 5′-3′ orientation, for RT-qPCR, cDNA amplification and the anneal/extension of large overlapping primers to create the stem loop secondary structure and YB-1 binding site mutants.

Luciferase	Sense	ggtcctatgattatgtccggttatgt
	Antisense	cgtcttcgtcccagtaagctatgt
GAPDH	Sense	cctctgacttcaacagcgacac
	Antisense	tgtcataccaggaaatgagcttga
TGF-β_1_	Sense	cctttcctgcttctcatggc
	Antisense	acttccagccgaggtccttg
β-actin	Sense	gacccagatcatgtttgagacctt
	Antisense	cagaggcgtacagggatagca
167	Sense	ccaagcttccttcgcgccctgggccatct
	Antisense	ccccatggctcctcggcgactccttcct
840	Sense	ccaagcttccttcgcgccctgggccatct
	Antisense	ccccatggcgagagcgcgaacagggctggtg
pGL3-77/105A	Sense	ccaagcttccttcgcgccctgggccatctccctccc acctccctccgcggagcagccagacagcgagggccc cggccgggggcaAAAAAAAAAAAAAAAAAAAAAAAA
	Antisense	ccccatgggctcctcggcgactccttcctccgctcc gggccgaggccggccccgcgggcggctcagagccgT TTTTTTTTTTTTTTTTTTTTTTTTTTTTtgcccccg
pGL3-92/105A	Sense	ccaagcttccttcgcgccctgggccatctccctccc acctccctccgcggagcagccagacagcgagggccc cggccgggggcaggggggacgccccgtAAAAAAAAA
	Antisense	ccccatgggctcctcggcgactccttcctccgctcc gggccgaggccggccccgcgggcggctcagagccgT TTTTTTTTTTTTTacggggcgtcccccctgcccccg
pGL3-92/98A	Sense	ccaagcttccttcgcgccctgggccatctccctccc acctccctccgcggagcagccagacagcgagggccc cggccgggggcaggggggacgccccgtAAAAAAAAc
	Antisense	ccccatgggctcctcggcgactccttcctccgctcc gggccgaggccggccccgcgggcggctcagagccgg gggggTTTTTTTTacggggcgtcccccctgcccccg
pGL3-99/105A	Sense	ccaagcttccttcgcgccctgggccatctccctccc acctccctccgcggagcagccagacagcgagggccc cggccgggggcaggggggacgccccgtccggggcaa
	Antisense	ccccatgggctcctcggcgactccttcctccgctcc gggccgaggccggccccgcgggcggctcagagccgT TTTTTTgccccggacggggcgtcccccctgcccccg
pGL3-50/56A	Sense	ccaagcttccttcgcgccctgggccatctccctccc acctccctccgcggagcagccAAAAAAAgagggccc cggccgggggcaggggggacgccccgtccggggcac
	Antisense	ccccatgggctcctcggcgactccttcctccgctcc gggccgaggccggccccgcgggcggctcagagccgg gggggtgccccggacggggcgtcccccctgcccccg

The altered residues in the mutants are denoted by capital letters.

A series of mutant luciferase reporter constructs were generated containing inserts of the TGF-β_1_ 5′UTR corresponding to nucleotides +1 to +167 with adenine substitutions to key residues in the stem loop secondary structure and the putative YB-1 binding sites (Figure 10). The inserts were generated by annealing two oligonucleotides (Table 1) with a central, overlapping, complementary region and then performing a primer extension with DNA Polymerase I, Large (Klenow) Fragment. The oligonucleotides contained the restriction sites *Hind*III and *Nco*I. The resultant inserts were digested with *Hind*III and *Nco*I and cloned into *Hind*III/*Nco*I digested pGL3-Control vector. The mutant luciferase reporter constructs were labelled as follows indicating the nucleotide position of the adenine substitution, pGL3-77/105A, pGL3-92/105A, pGL3-92/98A, pGL3-99/105A, and pGL3-50/56A.

To enable *in vitro* transcription the endogenous and mutated 167 nucleotide fragments were subcloned in pGEM-4Z vector (Promega), which contains a T7 RNA polymerase promoter site. The pGL3 luciferase reporter constructs containing the appropriate fragments were linearised with *Nco*I. The sticky ends were converted to blunt ends with DNA Polymerase I, Large (Klenow) Fragment. The fragments were then removed from pGL3 with *Hind*III and subcloned into *Sma*I/*Hind*III digested pGEM-4Z vector. The constructs were labelled as above indicating the nucleotide position of the adenine substitution, pGEM-4Z-77/105A, pGEM-4Z -92/105A, pGEM-4Z -92/98A, pGEM-4Z -99/105A, and pGEM-4Z-50/56A.

All plasmids were submitted for external sequencing analysis to ensure fidelity of amplification (www.dnaseq.co.uk).

### Transient Transfection and Luciferase Reporter Assay

Transfection was carried out in 24-well plates using Lipofectamine LTX and PLUS reagent (Invitrogen) according to the manufacturer's instructions. Cells were growth arrested in serum-free medium 4 hours prior to transfection with luciferase reporter constructs (pGL3) in combination with a renilla luciferase control plasmid (pRL-SV40) (Promega) at a ratio of 9∶1 respectively. Twenty-four hours after transfection the cells were lysed in passive lysis buffer (Promega). The firefly and renilla luciferase activity was measured by Dual-Luciferase Reporter Assay Kit (Promega) according to the manufacturer's instructions with a Fluostar Optima plate reading luminometer (BMG Labtechnologies, NC, USA).

### RT-qPCR for Luciferase mRNA

RNA was extracted 24 h following transfection of 6-well plates using a total RNA isolation kit (Agilent Technologies, Wilmington, USA). The samples were DNAse I treated to ensure no plasmid DNA contamination. cDNA was generated as previously described [10]. Primers to luciferase and GAPDH mRNA were designed using Primer3 (http://frodo.wi.mit.edu/primer3/input.htm) (Table 1.). The mRNA was quantified by RT-qPCR according to standard protocol using POWER SYBR® GREEN PCR Master Mix (Applied Biosystems, CA, USA) on a 7900HT Fast Real-Time PCR System (Applied Biosystems). The relative changes in gene expression were analysed by the 2^−ΔΔC^_T_ method [57].

### Electrophoretic Mobility Shift Assay (EMSA) and UV-Crosslinking

RNA probes for EMSAs and UV-Crosslinking were prepared by *in vitro* transcription of linearised pGEM-4Z vectors containing the endogenous or mutated 167 nucleotide fragments of the TGF-β_1_ 5′UTR. RNA probes internally labelled with ^32^P were generated using the T7 Riboprobe *in vitro* transcription kit (Promega). The DNA template was digested with DNase I, and the transcripts purified by illustra™ ProbeQuant™ G-50 Micro Columns (GE Healthcare, Buckinghamshire, UK) according to manufacturer's instructions. The EMSA mixtures contained 25,000 cpm of radiolabelled RNA and 1 µg recombinant or 5 µg total cell protein in a total volume of 20 µl containing 20 mM HEPES, 2 mM MgCl_2_, 5 mM dithiothreitol, 50 mM KCl, 40 µg/ml bovine serum albumin, 5% v/v glycerol, 1 U/µl rRNasin ribonuclease inhibitor, 50 µg/ml poly I∶C, 5 mg/ml heparin, and 100 µg/ml yeast tRNA. The binding reaction was performed at room temperature for 20 min followed by electrophoresis through a 6% non-denaturing polyacrylamide gel in 45 mM Tris-borate/1 mM EDTA, pH 8. The gels were dried and complexes detected by autoradiography.

The UV-Crosslinking mixtures contained 50,000 cpm of radiolabelled RNA and 1 µg recombinant or 5 µg total cell protein in a total volume of 10 µl containing 20 mM HEPES, 2 mM MgCl_2_, 5 mM dithiothreitol, 50 mM KCl, 40 µg/ml bovine serum albumin, 5% v/v glycerol, 50 µg/ml poly I∶C, 5 mg/ml heparin. The binding reaction was performed at room temperature for 20 min, followed by cooling on ice before UV-irradiating for 10 min using a Stratalinker XL1000 (Stratagene, La Jolla, CA, USA). The samples were treated with 100 µg/ml RNase A at 37°C for 1 h. An equal volume of non-denaturing loading buffer was added to the samples, which were heated to 95°C for 5 min followed by electrophoresis through a 10% SDS-polyacrylamide gel in 25 mM Tris-HCl, 192 mM Glycine, 0.1% SDS, pH 8.3. The gels were dried and complexes detected by autoradiography.

### Analysis of Efficiency of Translation

Polysome analysis was performed as previously described [11]. Approximately, 1×10^7^ growth-arrested cells per experiment were trypsinized, pelleted, and extracted in 1 ml of ice-cold lysis buffer consisting of 10 mM Tris-HCl, pH 8.0,150 mM NaCl, 1.5 mM MgCl_2_, 0.5% Nonidet P-40, 500 U/ml rRNasin ribonuclease inhibitor. Nuclei were removed by centrifugation at 3000 g for 2 min and the supernatant transferred to a new tube supplemented with 100 µg/ml cycloheximide, 1 mM PMSF, 10 mM DTT, and 0.5 mg/ml heparin, then centrifuged at 13000 g for 5 min to remove mitochondria and membrane debris. The supernatant was layered onto a 15% to 40% linear sucrose gradient containing 10 mM Tris-HCl, pH 7.5, 140 mM NaCl, 1.5 mM MgCl_2_, 10 mM DTT, 10 µg/ml cycloheximide, and 0.5 mg/ml heparin in a Polyallomer centrifuge tube (Beckman Coulter, CA, USA) and centrifuged using an SW41Ti rotor at 36,000 rpm for 2 h at 4°C. The gradient was fractionated into 22×0.5 ml fractions, each supplemented with 1% SDS, 10 mM EDTA, and 200 µg/ml proteinase K, and incubated at 37°C for 30 min to degrade endogenous nucleases. Subsequently, the fractions were mixed with phenol∶chloroform∶isoamyl alcohol (24∶24∶1) and the aqueous layer containing the RNA removed. A 5% aliquot of each fraction was analyzed by electrophoresis in a 3% agarose gel to ensure that the RNA was not degraded, and that the tRNA and rRNA species were appropriately distributed through the gradient. RNA was precipitated overnight at −20°C from the remainder of each fraction with 100% ethanol, 3 M sodium acetate and glycogen and washed once with 70% ethanol before air-drying. Fifty-percent samples of each fraction were run as a single large Northern blot, detected by autoradiography, and quantified by densitometry on a ChemiDoc (Bio-Rad, Hertfordshire, UK). Data are expressed as percentage of the total mRNA for that experiment in each fraction.

### Immuno-precipitation of mRNA/Protein Complexes (IP/RT-qPCR)

IP/RT-qPCR was performed according to the protocol of Peritz et al [58]. Cells cultured in 100 mm dishes were harvested in polysome lysis buffer (PLB) consisting of 100 mM KCl, 5 mM MgCl_2_, 10 mM HEPES, pH 7, 0.5% Nonidet P-40, 2 mM vanadyl ribonucleoside complex solution, 25 µl/ml protease inhibitor cocktail solution (Sigma), and passed through a 29-guage needle. The sheared lysates were centrifuged at 13,000 rpm at 4°C for 15 min to remove cell debris. The samples were split into 2×1 ml fractions and precleared with 50% protein A-agarose (Sigma) in PLB by incubation rotating at 4°C for 1 h. The fractions were centrifuged at 13,000 g for 1 min and the supernatants transferred to new tubes. 1 µg/ml polyclonal anti-YB-1 (YB-1-IP) antibody or non-immune rabbit-IgG (IgG-IP) were added to the fractions and incubated rotating at 4°C overnight. 50% protein A-agarose was added to the fractions and incubated rotating at 4°C for 4 h, followed by centrifugation at 13,000 g for 1 min and collection of the protein A-agarose beads. The protein A-agarose beads were washed repeatedly with PLB followed by a second round of washes with PLB containing 1 M urea. The protein A-agarose beads were resuspended in PLB containing 0.1% SDS and 30 µg proteinase K (Sigma) and incubated at 50°C for 30 min. An equal volume of phenol∶chloroform∶isoamyl alcohol (24∶24∶1) was added to the protein A-agarose beads and the aqueous layer containing the RNA removed. RNA was precipitated overnight at −20°C from the aqueous layer with 100% ethanol, 1 mg/ml yeast tRNA, 3 M sodium acetate. cDNA was generated as previously described [10] using a constant volume of RNA. Primers to TGF-β_1_ and β-actin mRNA were designed using Primer3 (http://frodo.wi.mit.edu/primer3/input.htm) (Table 1.). The mRNA was quantified by RT-qPCR according to standard protocol using POWER SYBR® GREEN PCR Master Mix (Applied Biosystems, CA, USA) on a 7900HT Fast Real-Time PCR System (Applied Biosystems). We assessed the input by measuring in parallel the mRNA in the IgG-IP extract and the YB-1-IP extract.
